# The processing of English regular inflections: Phonological cues to morphological structure

**DOI:** 10.1016/j.cognition.2008.06.011

**Published:** 2008-10

**Authors:** Brechtje Post, William D. Marslen-Wilson, Billi Randall, Lorraine K. Tyler

**Affiliations:** a Research Centre for English and Applied Linguistics, University of Cambridge, English Faculty Building, 9 West Road, Cambridge CB3 9DP, United Kingdom; b MRC Cognition and Brain Sciences Unit, 15 Chaucer Road, Cambridge, United Kingdom; c Centre for Speech, Language and the Brain, Department of Experimental Psychology, University of Cambridge, Downing Street, Cambridge, United Kingdom

**Keywords:** Speech processing, Morphology, Phonology, English inflection

## Abstract

Previous studies suggest that different neural and functional mechanisms are involved in the analysis of irregular (*caught*) and regular (*filled*) past tense forms in English. In particular, the comprehension and production of regular forms is argued to require processes of morpho-phonological assembly and disassembly, analysing these forms into a stem plus an inflectional affix (e.g., {fill} + {-ed}), as opposed to irregular forms, which do not have an overt stem + affix structure and must be analysed as full forms [Marslen-Wilson, W. D., & Tyler, L. K. (1997). Dissociating types of mental computation. *Nature, 387*, 592–594; Marslen-Wilson, W. D., & Tyler, L. K. (1998). Rules, representations, and the English past tense. *Trends in Cognitive Science, 2*, 428–435]. On this account, any incoming string that shows the critical diagnostic properties of an inflected form – a final coronal consonant (/t/, /d/, /s/, /z/) that agrees in voicing with the preceding segment as in filled, mild, or nilled – will automatically trigger an attempt at segmentation. We report an auditory speeded judgment experiment which explored the contribution of these critical morpho-phonological properties (labelled as the English inflectional rhyme pattern) to the processing of English regular inflections. The results show that any stimulus that can be interpreted as ending in a regular inflection, whether it is a real inflection (*filled–fill*), a pseudo-inflection (*mild–mile*) or a phonologically matched nonword (*nilled–nill*), is responded to more slowly than an unambiguously monomorphemic stimulus pair (e.g., *belt–bell).* This morpho-phonological effect was independent of phonological effects of voicing and syllabicity. The findings are interpreted as evidence for a basic morpho-phonological parsing process that applies to all items with the criterial phonological properties.

## 1 Introduction

Recent research into the neural and functional architecture of the human language system has been strongly influenced by the contrast between the regular and irregular past tense in English, which is regarded as a critical test case for discriminating competing claims about the organisation of the language system (Joanisse & Seidenberg, 1999; Marslen-Wilson & Tyler, 1997; Marslen-Wilson & Tyler, 1998; Marslen-Wilson & Tyler, 2003; Pinker, 1991; Pinker & Ullman, 2002; Plunkett & Marchman, 1993; Rumelhart & McClelland, 1986; Ullman 2004; Ullman et al., 1997). The central issue is whether the representation and processing of regular past tense forms, involving the combination of stems and affixes (e.g., *play* + /d/ = *played*), requires specialised cognitive and neural procedures which are not invoked by the unpredictable and idiosyncratic irregular forms (e.g., *buy*–*bought*, *hit*–*hit*, or *creep*–*crept*), where there is typically no overt combination of stem and affix.

The investigation of this issue has raised a set of more specific questions about the processing mechanisms involved in the cognitive analysis of regular and irregular forms in language comprehension (and production). Based on a number of neuropsychological and neuro-imaging studies, Marslen-Wilson, Tyler and colleagues argue for a distinction between lexical access processes that involve morpho-phonological decomposition, and those based on more direct access to stored forms (Marslen-Wilson & Tyler, 1997; Marslen-Wilson & Tyler, 1998; Marslen-Wilson & Tyler, 2003; Marslen-Wilson & Tyler, 2007; Tyler, Marslen-Wilson, & Stamatakis, 2005b; Tyler, Randall, & Marslen-Wilson, 2002b; Tyler, Stamatakis, Post, Randall, & Marslen-Wilson, 2005a; Tyler et al., 2002a). This emphasis on the morphological decomposition of regular inflected forms clearly allies this account with the “Words and Rules” (e.g., Clahsen, 1999; Pinker, 1999) and the procedural/declarative (e.g., Ullman, 2004) approaches, in distinction to non-decompositional, usually connectionist approaches, which deny the existence of separable stem and inflectional morphemes, and which argue instead that inflected forms are processed and represented as patterns of activation across pools of simple neuron-like processing units which share certain semantic, phonological and orthographic information (e.g., McClelland & Patterson, 2003; Rumelhart & McClelland, 1986). Unlike Pinker and colleagues, however, we do not assume that the presence or absence of grammatical morphemes implicates differences in the nature of mental computation. Our concern is with the functional architecture of the language processing system, not its underlying computational properties.

The Marslen-Wilson and Tyler morpho-phonological account was directly tested in a comprehension experiment with four nonfluent aphasics with a documented regular past tense deficit, using a speeded same–different judgment task (Tyler et al., 2002a). In this task, participants hear trials of matched pairs of spoken stimuli. Half of the trials are *same* pairs in which the first word is repeated (e.g., *filled–filled*), and half are *different* pairs with a minimally different first and second word (e.g., *filled–fill*). Participants decide as quickly and as accurately as possible whether the two members of a pair sound the same or different. The effects of phonological complexity were controlled by including monomorphemic words and nonwords that were matched for form on a one-to-one basis to the regular and irregular pairs (e.g., pseudo-regular *jade–jay* and nonword *kade–kay* for real regular *played–play*). In a further condition, controlling for possible segmentation effects, the first and the second word again differed in the removal of the final phoneme of the first member of the pair (e.g., *claim–clay*), but here the final phoneme was not a possible inflectional affix.

There were two important aspects to the results. The first was that the performance of patients with past tense deficits was most impaired for the real regular pairs, with significantly slower responses than to matched conditions (such as pseudo-regulars and nonword regulars). The second main result – and the stimulus for the research reported here – was that overall, the patients were substantially impaired for *all* conditions that contained a potential regular inflectional affix. Thus, although performance was poorest for the real regulars (mean reaction time of 1420 ms and error rate of 31%), it was also poor both for the pseudo-regulars (RT of 1252 ms and error rate of 25%) and for the nonword regulars (RT of 1244 ms and error rate of 22%). Performance was much less impaired, and closer to normal levels of accuracy, for the control pairs such as *claim–clay* (RT of 1044 ms and error rate of 5%), where the first member of the pair did not end in a potential affix. A similar grouping of responses to real regulars, pseudo-regulars and nonword regulars, in distinction to morphologically simple pairs of the *claim–clay* type, was also observed in a subsequent neuro-imaging study, where unimpaired young adults performed the same–different task on the same types of material in an event-related fMRI study (Tyler et al., 2005a).

The hypothesis we explore here is that this commonality between the three critical regular past tense conditions (real, pseudo, nonword) reflects their common morpho-phonological properties – i.e., that they all share specific phonological features that are diagnostic of the presence of a potential inflectional suffix, and will therefore place specific demands on the neural and functional machinery underlying the perceptual processing of spoken words in English. These diagnostic phonological features, which we label the English *inflectional rhyme pattern* (IRP), have two components: the presence of a word-final coronal consonant (i.e., any sound articulated with the tip or blade of the tongue raised towards the teeth or the alveolar ridge, such as /d, t, s, z/), and the agreement in voice between the final coronal consonant and the segment that precedes it. Thus, the sequence *passed* /pa:st/ is a potential combination of a verbal stem *pass* /pa:s/ with an inflectional suffix, because it ends with the unvoiced coronal consonant /t/, and this agrees in voice with the preceding unvoiced segment /s/. The same applies to the pseudo-regular word *fast*, which is potentially analyzable as the (nonexistent) verb stem /fa:s/, plus the inflectional morpheme /t/. The same, furthermore, would also hold for the nonword sequence *nast*, also potentially analyzable as /na:s/ plus /t/.

The presence or absence of these diagnostic features (the IRP) will lead a spoken lexical input to interact differentially with the machinery of lexical access and linguistic interpretation (cf. Marslen-Wilson & Tyler, 2007). This machinery, broadly speaking, is concerned on the one hand with the mapping of phonological inputs onto stored lexical representations, and on the other with the extraction and interpretation of grammatical morphemes (and other cues to structure). The successful functioning of this system requires the appropriate segmentation of speech inputs into stems and different types of affix. When an input such as /pa:st/ is encountered, corresponding to the past tense form *passed*, the system needs both to access the semantic and syntactic properties associated with the stem {pass} and to extract the processing implications of the presence of the grammatical morpheme {-t}.

A critical claim about the functioning of this system – motivated in particular by the effects for nonword regulars in the earlier neuropsychological and neuro-imaging studies (Tyler et al., 2002a; Tyler et al., 2005a) – is that the attempt at segmentation into stem and affix is automatically triggered by any input that has the critical diagnostic properties. Whenever the system encounters a candidate string that ends with a coronal consonant that agrees in voice with its preceding segment, this will always have to be evaluated both as a monomorphemic input and as a verbal or nominal stem with an accompanying inflectional affix. This seems to be forced by the pervasive ambiguity of possible inflected inputs. An input like /peIst/ may be either the monomorphemic form *paste* or the past tense of *pace*; /treId/ could be the past tense of the stem *tray*, and so forth. The system cannot decide in advance which strings with the appropriate properties are inflected forms or not – even if, in a case like *trade*, the stem in question may never be used as a verb.

This predicts that listeners should make slower responses whenever they are asked to make same–different judgments where at least one member of the pair ends with the characteristic inflectional rhyme pattern, relative to control pairs which deviate from this pattern – for example, pairs like *tent–ten* (where the first member of the pair ends in a coronal which does not agree in voicing with the preceding segment) or *clamp–clam* and *bark–bar* (where the first word ends in labial or velar consonants). These are less complex to process because they can only be interpreted as full forms, and will not engage morpho-phonological segmentation mechanisms that generate additional lexical complexity and competition. The purpose of the research reported here, therefore, is to explore this specifically morpho-phonological hypothesis about the nature of the processing operations associated with the presence of regular inflectional morphology in English.

We need to consider this hypothesis, however, in the context of a widely held competing view, which argues that patients’ difficulties with regular past tense inflections do not reflect the morphological or morpho-phonological properties of inflected words, but rather purely phonetic and phonological factors relating to the perceptual complexity of the forms in question. These are the single mechanism connectionist models, where there is no independent representation of morphology, either regular or irregular. The currently most prominent of these models has been developed by Joanisse and Seidenberg (1999), claiming that differential effects for regulars and irregulars can be modelled in a single undifferentiated network without reference to morphological features, by exploiting the statistical regularities in the variation of semantic and phonological overlap between stem and past tense form that distinguish regulars and irregulars. This approach makes the strong prediction that any apparently morpho-phonological effects for regular inflected forms are in fact primarily phonological in nature (cf. Bird, Lambon Ralph, Seidenberg, McClelland, & Patterson, 2003; McClelland & Patterson, 2003). Regulars are argued to be more difficult to process than irregulars because they have “greater articulatory complexity and perceptual subtlety” (McClelland & Patterson, 2003, p63). The poorer performance of nonfluent patients on regular forms is therefore primarily attributed to general problems in phonological processing, and not to any specifically morphological deficit (e.g., Bird et al., 2003; cf. Tyler et al., 2002b, and Marshall & Van der Lely, 2006, for a recent study with children).

Although notions of phonological complexity and perceptual salience are not fully defined in the relevant single mechanism publications, they can be taken to refer to characteristic phonological properties of regular inflected forms – in particular the structure of the final consonant cluster, and the types of phoneme involved. Since the inflection is realised as /t/ or /d/ except when the final consonant is already a /t/ or /d/ (as in forms like *greeted* or *sighted*), this often leads to unusual final consonant clusters like /spt/ in *clasped* (cf. Marshall & van der Lely, 2006). At the same time, as noted previously, the inflectional morpheme always ends in a coronal, which may have a special status linguistically (Paradis & Prunet, 1991). Coronals are the most common phonemes cross-linguistically (Maddieson, 1985). Moreover, they may be perceptually less salient than labials (like /p/) and dorsals (like /k/; Hume, Johnson, Seo, & Tserdanelis, 1999, for stops in Korean and English), they tend to be replaced by velars or alveolars in speech errors (Stemberger, 1991), and they have been found to be more susceptible to regressive place assimilation (Jun, 1995, reported in Kochetov, 2004). There is no clear evidence, however, that coronality itself selectively affects the phonological processing of regular past tense forms.

In the current experiment, we will evaluate the processing implications of a number of phonological and morpho-phonological properties of regular inflected forms in English, taking care to control closely for phonological complexity, defined as the type of CV sequence that makes up a particular word form. The further organisation of the experiment is laid out in the following section.

### 1.1 The inflectional rhyme pattern: Coronality and voicing

The primary set of contrasts explores the significance of the different components of the diagnostic rhyme pattern that indicate the presence of a potential word-final inflectional morpheme. Taking a set of real regular past tense items (*filled–fill*, *blessed–bless*) as a form of baseline, these are compared with three sets of morphologically simple real words, as well as with matched sets of nonwords, as shown in Table 1.

The first comparison set of real words, as in previous experiments, is a set of pseudo-regular forms like *mild–mile* or *crest–cress*. These [+Coronal, +VoiceAgreement] materials share the inflectional rhyme pattern, and have an embedded real stem, but the full form is not itself an attested past tense form. We expect slower processing of these forms relative to uninflected forms that do not have the rhyme pattern, just as for the real regulars, because the presence of the rhyme pattern should trigger the same morpho-phonological segmentation and evaluation processes. These processes will activate, for example, the spurious lexical candidate *cress*, when the first word *crest* is heard, which should both increase processing load for *crest*, and potentially interfere with the same–different decision to the second word, *cress*.

The second comparison set, containing pairs like *start–star* or *tent–ten*, retains the coronal ending, but violates the second component of the inflectional rhyme pattern, since this final consonant does not agree in voice with the preceding consonant – if *ten* had a past tense, it would have to be *tenned* (/tend/, homophonous with *tend*). If the presence of a word-final coronal intrinsically causes perceptual difficulties, then responses should also be slower for these [+Coronal, −VoiceAgreement] materials. If it is the presence of the inflectional rhyme pattern that is critical, then these materials should be treated as monomorphemic, and therefore less complex than either the regulars or the pseudo-regulars.

The third set of words moves further away from the inflectional rhyme pattern by replacing the final consonant with a noncoronal segment (either a labial /p/, as in *clamp–clam*, or a dorsal /k/, as in *milk–mill*). These [−Coronal, −VoiceAgreement] materials violate both components of the inflectional rhyme pattern, and should be treated as monomorphemic full forms in the same way as the [+Coronal, −VoiceAgreement] stimuli in the previous condition. However, if it is the presence of a coronal consonant that is critical, on a perceptual difficulty account, then performance should be improved here, relative to the [+Coronal] conditions.

In order to neutralise the phonological complexity issue, all three of these sets of words will be closely matched to each other, and to the real regulars, in their CV structures, covering both word onsets and word offsets. If there are differences between conditions, these should not reflect differences in complexity. Note that this means that highly complex regular past tense forms, ending in a CCC sequence (as in *clasped* or *asked*), are not included here as part of the main experiment, since they cannot be matched across the other real word conditions.

The three sets of monomorphemic comparison conditions were accompanied by three matched sets of nonwords – a pseudo-regular [+Coronal, +VoiceAgreement] set, as in *minned–min* /mind/ /min/ or *stessed–stess* /stest/ /stes/, a coronal noninflectional [+Coronal, −VoiceAgreement] set, as in *rint–rin* /rint/ /rin/ or *lart–lar* /la:t/ /la:/ and a noncoronal [−Coronal, −VoiceAgreement] set, as in *plamp–plam* /plæmp/ /plæm/ or *tulk–tul* . Again, the morpho-phonological account predicts more complex processing for the pseudo-regular set than for the other two, on the assumption that the presence of the inflectional rhyme pattern will trigger segmentation attempts that generate a pseudo-stem, such as *min* or *stess*, potentially disrupting same–different judgements to the actual second member of the pair. On the perceptual difficulty account, the [−Coronal, −VoiceAgreement] stimuli should contrast with the first two sets which both contain word-final coronal consonants. All three sets are matched to each other, and to the real regulars, in their CV structure.

Two further contrasts complement this main set of materials, while also expanding the variety of materials the participants are exposed to. The first of these, using the *s* inflection, expands the coverage of this research beyond the set of past tense inflections, allowing us to examine the generality of the claims being made here for the influence of the inflectional rhyme pattern.

The regular *s* inflection in English, used to mark noun plurals (as in *cats–cat* or *yards–yard*) and the third person present tense (as in *lick–licks* or *begs–beg*), observes the same diagnostic constraints as the past tense inflection. The inflection itself, whether realised as an /s/ or a /z/, is a coronal consonant, and it must agree in voice with the preceding segment – a form like *sparse* cannot be interpreted as the plural of *spar*. The difference with the regular past tense forms is the manner of articulation of the final consonant; /s/ and /z/ are fricatives, while /t/ and /d/ are stops. If the inflectional rhyme pattern has the same processing consequences here as for the past tense inflection, then we expect present tense and plural forms in /s/ and /z/ to pattern with the real and pseudo past tense forms in /t/ and /d/, rather than with the [+Coronal, −VoiceAgreement] and [−Coronal, −VoiceAgreement] sets, such as *tent–ten* and *milk–mill*, which do not conform to the inflectional rhyme pattern. The same should hold, for the same reasons, for nonword pairs like *pakes–pake* /peiks/ /peik/ or *dags–dag* /dægz/ /dæg/, relative to the parallel past tense nonword sets.

English regular inflection also includes two cases which do not obey the inflectional rhyme constraint, and which are both syllabic, as opposed to the cases we have considered above. These are the syllabic past tense allomorph /id/ (e.g., *folded–fold*), and the progressive aspect morpheme , which we include here primarily to give full coverage of English inflection, as well as increasing the variety of materials the participants are exposed to. Although the syllabic past tense allomorph /id/ ends in a coronal just like the nonsyllabic past tense allomorphs /d/ and /t/, it does not show voicing agreement with the rhyme. Progressive  does not share any phonological features with the nonsyllabic allomorphs. There is not a clear prediction here for the same–different task. In the absence of the rhyme diagnostics, we would not expect to see the slow down in response times that we anticipate for the monosyllabic inflections. At the same time, it is possible that the syllabic nature of the inflection makes the same–different judgments highly perceptually salient, which may be reflected in faster response times.

## 2 Methods

### 2.1 Design

We examined the contribution of morphological and phonological factors to regular past tense processing by systematically manipulating, relative to a baseline set of past tense forms (condition 1 in Table 1 above): (i) coronality and voicing (inflectional rhyme pattern present in condition 2; absent in conditions 3 and 4), (ii) inflectional paradigm (present tense and plural in conditions 5 and 6), and (iii) syllabicity (syllabic allomorphs in conditions 7 and 8). The items are listed in Appendix A.

### 2.2 Materials

There were 48 trials per condition, consisting of 24 *different* pairs with a minimally different first and second word (or nonword) which were also used as *same* pairs in which the first word was repeated as the second word. Eight of the 14 conditions were real word conditions and six were nonword conditions. The nonword items were mostly derived from the real words in the corresponding conditions so as to maximise their phonological similarity (sharing as many phonemes in onsets, nuclei and codas as possible). Except for items in real inflected conditions, the word pairs were semantically unrelated. All items were based on matched monosyllabic and monomorphemic stems, to avoid any confound between morpho-phonological decomposition of inflections and other morphological or phonological processes. All monosyllabic conditions were matched for the phonological complexity of the first member of each test pair, with complexity defined here as CV structure. This covered both onsets and codas across all the relevant conditions. If, for example, the regular past tense set contained the form *prayed*, with the CV structure CCVC, then a form with the same CV structure would occur across the other conditions. For all same–different pairs, the second word of the pair would have the same CV structure but without the final segment. For the syllabic conditions, the CV structure of the stem of the first word in the pair was used for matching instead of the whole word form (e.g., *greeted–greet* was matched with *prayed–pray*).

Since the experiment contained monosyllabic and bisyllabic items in different conditions, the duration of the second word varied significantly between conditions (see Table 2; [*F*(13, 648) = 11.20, MSE = 137,899, *p* < .001]). A second factor that played a role in the duration of the second word was the voicing of the final consonant (Gimson, 1961). In English, vowels that precede a voiceless consonant are shorter than vowels which precede voiced material (e.g., *plate* is much shorter than *played*; e.g., Wiik (1965)). In our experiment, since the monomorphemic conditions in /t/, /k/ and /p/ all ended in voiceless consonants, while the morphologically complex forms and the pseudo past contained both voiced and voiceless codas (12/24 voiced codas for real past, present and plural, and 16/24 voiced codas for the pseudo past), the second word in the word pairs in the latter conditions are on average longer than in the former (see Table 2). The conditions could not be matched for voicing, because the set of pseudo past forms is too small to be limited to the subset with voiceless codas. The alternative of voicing half of the items in the nonpast condition in /t/ was not a possibility, since this would have led to voicing agreement in the rhyme, turning them into pseudo past or real past items (e.g., voicing final /t/ in *trait* gives *trade*). This means that both voicing and duration of the second word have to be taken into account in the reaction time analyses.

We controlled as far as possible for form class ambiguity. Items used in verbal contexts were generally more frequent as verbs, even if a noun form existed and items used in nominal contexts were more frequent as nouns, even when a homonymic verb existed. In addition, subject to the above constraints, we matched the conditions as closely as possible for lemma frequency, word form frequency, familiarity and imageability (see Table 3; based on the CELEX lexical database (Baayen, Piepenbrock, & Gulikers, 1995), the MRC Psycholinguistic data base (Wilson, 1988), and our own data base of locally collected information from paper-and-pencil rating tasks with minimally 15 participants). One-way analyses of variance, carried out for each “nuisance” variable with the factor condition (8 levels), nevertheless showed a significant main effect for each variable, with the exception of familiarity of the second word in the word pair.^1^

The stimuli were recorded on DAT tape in a sound-attenuated booth, and digitised at 22.05 kHz for further processing. The first item in each pair was spoken by a male speaker, and the second by a female speaker to ensure that the judgments were not made on the basis of the low-level acoustic or phonetic properties of the test pairs. Both speakers were native speakers of British English.

### 2.3 Procedure

The pairs were presented in a single-version experiment in pseudo-random order in four sessions of 184 stimuli, each introduced by 4 dummy stimuli, and with equal numbers of pairs from each condition in each session (108 real words and 72 nonwords). Numbers of *same* and *different* stimuli were also balanced (90 of each per session), and at least one session intervened between repetitions of an item as a *same* or a *different* stimulus. The experiment began with a practise session (24 items), and each session was followed by a short break. The experimental software package DMDX (Forster & Forster, 2003) was used for the presentation of the stimuli. Participants were tested in quiet conditions, using a two-button response box, and wearing headphones. They would first hear the inflected form or its equivalent, spoken by the male voice, and after a 100 ms delay, they would hear the second item, spoken by the female voice. The intertrial interval was 850 ms, and the time-out was set at 3 s. The participants were asked to press the button labelled *same* when the two items sounded the same, and *different* when they sounded different. The experiment took about 45 min.

### 2.4 Participants

We tested 20 participants, 5 men and 15 women, from the subject pool of the Centre for Speech, Language and the Brain. They were all native speakers of English, aged between 18 and 25, and had no known hearing deficits. The participants were paid a small fee.

## 3 Results

### 3.1 Reaction times

One item was removed from the analysis because of a very high error rate (85%), and nine items were lost because of programming error (1.25% of items).^2^ The RT data, which were only collected for correct responses, were then inverse transformed to reduce the effects of outliers (Ratcliff, 1993). In the subject analysis (F1), the data were averaged over items, and in the item analysis (F2), over subjects. The mean reaction times and error proportions are shown for each condition in Table 4.

As a first examination of the data, we conducted an overall analysis of variance to see if there were differences between conditions and judgment types. There were two repeated measures in the subject analysis (condition with 14 levels and judgment type with two levels: same and different). In the item analysis, there was one repeated measure (judgment type) and one independent measure (condition). The results show a significant effect of condition [*F*1(13, 247) = 53.42, *p* < .001; *F*2(13, 312) = 8.41, *p* < .001], and a marginal effect of judgment type in the items analysis only [*F*1(1, 19) = 1.09, *p* = .310; *F*2(1, 312) = 3.41, *p* = .07]. In addition to the main effects there was a significant interaction between condition and judgment type [*F*1(13, 247) = 6.51, *p* < .001; *F*2(13, 312) = 2.06, *p* < .05].^3^ This is because *same* items are not always responded to faster than *different* items, even though overall, *same* items are responded to 20 ms more slowly than *different* items (see Table 4 and Appendix B; this interaction is explored further in the regression analyses reported below).

The overall effect of condition justifies further analyses to explore the principal factors of interest *–* coronality and voicing, inflectional paradigm (reflected by manner of articulation) and syllabicity. At the same time, we needed to evaluate whether any of the “nuisance” variables affected reaction time, especially since not all of these could be fully matched across conditions (see Tables 2 and 3 above). Correlation analyses showed that none of these variables, except for the duration of the second word [*r*(662) = −0.49, *p* < .001], did in fact correlate with RT.^4^

We also tested for a possible confound, raised by a reviewer, with the diphone frequency of the final phonemes in the word pairs. This might have affected reaction times if participants simply relied on how dissimilar the final phonemes were to make their judgements. Responses would be quickest for *same* items, where the words of the pair are identical. For *different* items, however, less frequent combinations of consonants might be harder to process than more frequent ones, resulting in relatively slower reaction times. Calculations of the logged frequencies of the final diphones of word 1 and word 2 in the stimulus pairs as well as their ratio (using CELEX wordform frequencies, Baayen et al., 1995), showed that these did differ significantly between conditions. Correlational analyses, however, showed that diphone frequency did not correlate with RT in the experiment: word 1 *r*(379) = −.05, *p* = .31, word 2 *r*(379) = −.08, *p* = .13; ratio diphone frequency word 1/word 2 *r*(379) = −.08, *p* = .14.

In the main analysis reported here, using regression techniques, we included the duration of the second word as a continuous regressor, in addition to the binary regressors which represented the factors of interest. These were (1) morphological status: items that ended in a real inflectional affix versus items that were monomorphemic (including the pseudo past tense forms), (2) word type: real words versus nonwords, (3) rhyme pattern: items that are compatible with an inflection (including pseudo past forms) versus all other items, (4) place of articulation: items that end in a coronal consonant versus all other items, (5) voice: items that end in a voiceless consonant versus items that end in a voiced consonant (including bisyllabic past and progressive forms), (6) syllabicity: mono- versus bisyllabic forms, and (7) manner of articulation: forms that end in a stop (including bisyllabic past tense forms) versus all other forms. We also included all interactions with judgment type (*same* or *different* item), because of the presence of the judgement type/condition interaction in the overall analysis of variance. The most important question here was whether the rhyme pattern effect varied as a function of the judgement required. The results are summarised in Table 5.

The duration of the second word, the rhyme pattern, syllabicity and judgment type all contributed significantly to response times in our experiment but morphological status, voice, word type, and place and manner of articulation do not. The results also show (Table 5, level 2) that judgment type does not interact significantly with rhyme pattern (for estimated marginal means for same and different items in each condition see Appendix B). Although a number of other factors did interact with judgement type, none of these seem to bear significantly on the main questions at issue here.^5^

**Fig. 1:**
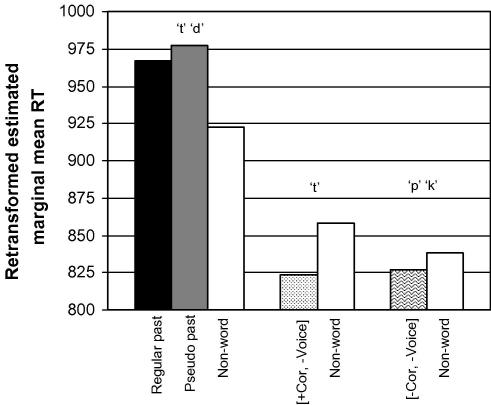
Harmonic estimated marginal mean reaction times for the conditions testing coronality and the pattern of the rhyme (*different* items only).

For ease of visualisation, Fig. 1 shows the effect of the rhyme pattern in terms of the relevant experimental conditions. The mean reaction times in the figure have been corrected for the effect of the duration of the second word by calculating estimated marginal means in an analysis of variance with the fixed factor condition (14 levels) and the duration of the second word as a covariate [condition: F(13, 313) = 12.66, p < .001; duration word 2: F(1, 313) = 109.53, p < .001] (see Appendix B for *same* items).

The cluster on the left-hand side represents real, pseudo and nonword regular past forms which have the rhyme pattern, and they clearly have longer reaction times than the other clusters which represent uninflected forms that do not have the rhyme pattern (all *p* < .01 in Bonferroni post hoc comparisons, except for nonword /t/ /d/ versus nonword /t/ *p* > .10). This effect occurs irrespective of coronality, morphological status and word type.

**Fig. 2:**
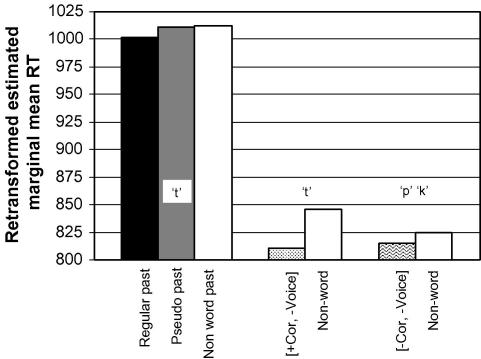
Harmonic estimated marginal mean reaction times for the voiceless items in the conditions testing coronality and rhyme pattern (*different* items only).

Since voicing affected reaction times in the regression analysis (although only for *different* items), and, as mentioned in Section 2 above, the conditions were not matched for voicing, the question arises how this affects the morpho-phonological effect of the rhyme pattern. Fig. 2 shows the mean values for the same conditions when all voiced items have been excluded, estimated in an analysis of variance with the fixed factor condition (10 levels) and the duration of the second word as a covariate [condition: *F*(9, 147) = 12.42, *p* < .001; duration word 2: *F*(1, 147) = 46.36, *p* < .001]^6^ (see Appendix B).

The figure shows that voicing has an effect, with increased response times in real, pseudo and nonword past items when voiced items are removed from these conditions. As a result, the differences between the conditions with and without the rhyme pattern are even larger than those estimated in Fig. 1, corresponding to a robust morpho-phonological effect (all *p* < .001 in Bonferroni post hoc comparisons). This enhanced effect indicates that the lack of matching on voicing across conditions in fact biased the results against our morpho-phonological hypothesis, rather than creating a confound.

**Fig. 3:**
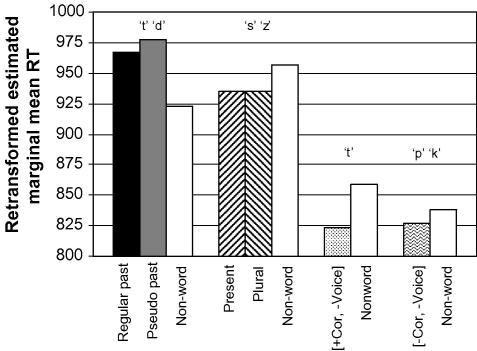
Harmonic estimated marginal means for the conditions testing manner of articulation (inflectional paradigm), coronality and rhyme pattern (*different* items only).

Fig. 3 illustrates that the effect of the rhyme pattern is not restricted to real and pseudo past tense forms, but also applies to the *s* inflection forms. The means in this figure were estimated in the same covariate analysis used for Fig. 1 (see Appendix B). Since manner of articulation did not contribute to reaction times, we can conclude that the morpho-phonological effect of the rhyme pattern appears to extend to all monosyllabic inflections in the experiment (all *p* < .01 in Bonferroni post hoc comparisons, except for nonword /t/ versus nonword /s/ and /z/, and nonword /t/ versus nonword /t/ and /d/, which were not significant).

**Fig. 4:**
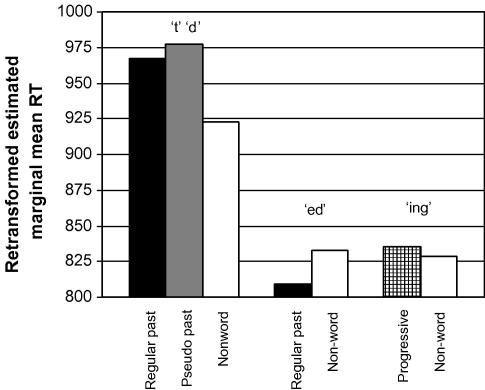
Harmonic estimated marginal mean reaction times for the conditions testing syllabicity (*different* items only).

Finally, Fig. 4 illustrates the purely phonological effect of syllabicity on response times when word 2 duration is taken into account (again estimated in the covariate analysis used for Fig. 1; see Appendix B). Syllabic inflections are responded to faster than nonsyllabic ones (all *p* < .05 in Bonferroni post hoc comparisons, except for pseudo past versus progressive  *p* > .10, and nonword /t/ and /d/ versus nonword /Id/ *p* > .10).

### 3.2 Errors

Although the error rates were very low, the same set of analyses was carried out on the arcsine transformed proportion correct responses. The subjects and items analyses showed a significant effect of condition [*F*1(13, 247) = 5.06, MSE = 0.251, *p* < .001; *F*2(13, 312) = 1.82, MSE = 0.144, *p* < .05], and an interaction between judgment type and condition [*F*1(13, 247) = 2.99, MSE = 0.151, *p* < .001; *F*2(13, 312) = 2.34, MSE = 0.186, *p* < .01], but no main effect of judgment type [*F*1(1, 19) = 2.32, MSE = 0.289, *p* = .14; *F*2(1, 312) = 1.42, MSE = 0.113, *p* = .23].

None of the nuisance variables (listed in Tables 2 and 3 above) correlated with error proportion.^7^ Therefore, we only included our factors of interest in the regression analysis, together with judgment type: (1) morphological status, (2) rhyme pattern, (3) syllabicity, (4) voicing, (5) manner of articulation, (6) place of articulation, and (7) word type. Rhyme pattern (*β* = −0.141, *p* < .05) and judgment type (*β* = 0.226, *p* < .001) contributed to the error scores, and there were interactions between judgment type and rhyme pattern (*β* = −0.299, *p* < .001), judgment type and word type (*β* = −0.130, *p* < .05), judgment type and morphological structure (*β* = 0.159, *p* < .05), and judgment type and voicing (*β* = 0.194, *p* = .001; nonsignificant regressors: morphological structure *p* = .56, syllabicity *p* = .33, voicing *p* = .14, manner *p* = .88, place p = .50, word type *p* = .70).

The major effect of the rhyme pattern indicates that all potentially inflected forms, such as *filled–fill*, *fails–fail* and *mild–mile*, are more likely to be heard as *same* items when they are actually different, compared to unambiguously monomorphemic items like *saint–sane* and *bank–bang*. This is consistent with the view that the morpho-phonological properties of the incoming speech sounds automatically trigger an attempt at segmentation into a stem and an affix, but only when the rhyme pattern signals that it is compatible with an inflection.

Finally, we established that although reaction times did correlate with error proportion [*r*(379) = 0.31, *p* < .001), there was no speed–accuracy trade-off, since faster reaction times tended to coincide with lower error rates.

## 4 Discussion

A clear pattern of results emerges from this experiment, showing that the processing of regular English inflections is influenced by morpho-phonological as well as phonological factors. We hypothesised that the neural and functional mechanisms involved in the processing of spoken words in English are differentially engaged by monomorphemic forms and by real and pseudo-inflected forms which show the critical diagnostic properties of morphologically complex inflectional forms. These diagnostic properties (the IRP) are voicing agreement in the syllable rhyme in combination with a coronal place of articulation for the final consonant of the (pseudo-)inflected form (e.g., *filled*, *fails, meals* or *mild*, but not *saint* or *bank*). When the IRP is present, and since the perceptual system cannot decide on the basis of acoustic–phonetic information alone whether a form does in fact bear an inflection, the presence of the IRP should trigger automatically an attempt at segmentation into a stem and an affix. This would not be triggered by unambiguously monomorphemic forms. We therefore predicted a morpho-phonological effect reflected in a difference between items that showed the diagnostic rhyme pattern and those that did not, regardless of their actual morphological status.

Our findings support this hypothesis. In a same–different judgment task, we found elevated judgment times for potentially inflected items compared to items that could not be interpreted as inflected forms.^8^ The difference between the potentially inflected and the uninflected forms in /t/ and /d/ shows that coronality only has an effect when it combines with voicing agreement in the rhyme, but not when it occurs on its own. The findings for the pseudo- and nonword inflections confirm that the rhyme pattern itself is the critical feature, rather than the actual morphological status of the item, and that segmentation must be automatic, since reaction times are comparable for any item that shows the diagnostic pattern, including meaningless strings of sounds and real words that are not actually inflected. This also includes present and plural inflections which also combine coronality and voicing agreement in their rhymes, but do not have the same place of articulation as the regular past tense (i.e., fricative /s/ and /z/).

In addition to the morpho-phonological effect, we found effects for two purely phonological factors that are independent of the morphological status of the word, for which we had made no predictions. The results show that when the past tense morpheme is realised as a syllable, participants are faster to make same–different judgments than when it is realised as a single segment. This effect of syllabicity extends to the progressive inflection , where reaction times were comparable to the syllabic past, and to the matched nonword conditions, which behaved just like the real word syllabic items.

This across-the-board syllabic effect is likely to reflect the greater perceptual salience of syllabic inflections (or pseudo-inflections), even before the added syllable is heard. Polysyllabic forms provide additional early phonological cues to the presence or absence of extra material at the end of the word, giving a strong indication of whether both words in the stimulus pair are the same or different. A strong early cue is the shortening of the first syllable – for example in *melted* relative to *melt* (Klatt, 1976; Lehiste, 1972) *–* which has been shown to affect lexical access processes in spoken word recognition (Davis, Marslen-Wilson, & Gaskell, 2002). Other early cues could involve changes in metrical structure and in syllabification.

The second phonological factor was voicing of the final segment. Overall, items which end in a voiced consonant, such as *raised – raise*, are responded to more quickly than items that end in a voiceless consonant like *raced – race*. Listeners pick up on cues to the presence or absence of a final consonant more quickly in the voiced items, possibly because cues to voicing become available early in the speech signal (e.g., Hawkins & Nguyen, 2004; Peterson & Lehiste, 1960). Such cues are exploited online to identify a word before the consonant in question is fully articulated (Warren & Marslen-Wilson, 1987; Warren & Marslen-Wilson, 1988), although it is not clear why the same cues could not be used to determine that a voiceless item was being heard.

The voicing effect is independent of the morpho-phonological effect, although it introduced a bias against our hypothesis in the data. As explained in Section 2 above, the conditions did not have equal numbers of items with voiced and voiceless final consonants. Since the (pseudo)inflected items had both voiced and voiceless endings, while uninflected items only had voiceless final consonants, the faster response times for the voiced items in the (pseudo)inflected conditions speeded up responses relative to the uninflected conditions. As a result, when voicing was eliminated as a factor, the morpho-phonological effect became even stronger, further increasing the difference between monomorphemic and morphologically complex items.

## 5 Conclusion

The segmentation of regular and pseudo-regular forms places specific demands on the neural and functional mechanisms involved in speech processing. We propose that the neuro-cognitive machinery underlying perceptual processing of spoken words in English involves both lexical access and automatic interpretative processing of the grammatical properties of the incoming words, where the extraction and interpretation of grammatical morphemes is triggered by morpho-phonological cues. This view is best accommodated in an account in which segmentation of incoming speech sounds into stems and affixes is distinguished from direct mapping between phonological forms and lexical representations.

This decompositional account of the processing of regular inflections in English (Marslen-Wilson & Tyler, 2007), invoking a core left fronto-temporal neural substrate, would explain the results of the neuropsychological study discussed earlier (Tyler et al., 2002), in which patients not only had processing difficulties with regular past tense forms, but showed diminished performance across the board for items that show the critical morpho-phonological properties of inflected forms. It would also accommodate findings from priming experiments with patients with a regular past tense deficit, where regularly inflected forms did not prime either morphologically related forms (*jumped – jump*; Tyler, de Mornay-Davies, et al., 2002a) or semantically related forms (*jumped – leap*; Longworth, Marslen-Wilson, Randall, & Tyler, 2005). If the patient’s morpho-phonologically triggered processing of these forms is disrupted, this would impair access to stored lexical representations of underlying stems. The morpho-phonological account also provides an alternative interpretation of some of the findings of the neuropsychological study of Bird et al. (2003), who included two conditions that contrasted in voicing agreement (*an – and* versus *an – ant*) in a similar manner to the pseudo-inflected and [+Coronal, −Voice] conditions of the present experiment. Patients’ performance was worse for the former, which exhibited the critical morpho-phonological properties of inflections, than for the latter, which did not.

In fact, if the pattern of the rhyme is as strong a cue as the present findings suggest, then the processing of irregular past tense forms that have the IRP, like *slept*, should initially resemble that of regulars like *stepped*, since the perceptual system is blind to the morphological composition of the incoming signal. The change in vowel quality in irregular past forms would still provide an early cue to the presence of an irregular rather than a real regular past tense form, but the morpho-phonologically triggered attempt at decomposing the incoming string might still slow down response times relative to “irregular irregulars” like *took*. This type of morpho-phonologically triggered processing may have contributed to Joanisse and Seidenberg’s recent fMRI findings, using a verb production task, which showed different activation effects in both left and right frontal regions for regulars than for irregulars, but similar activation patterns to regulars for a subset of pseudo-regular irregulars of the *slept* type (Joanisse & Seidenberg, 1999).

This is not to say that there is no contribution from phonology in the processing of inflected forms. On the contrary, our findings for voicing and syllabicity confirm that phonological factors do play an important role in the ability to process inflected words in English, complementing previous findings about factors such as phonotactic probabilities, various semantic properties, and stem and word form frequency, which have also been shown to impinge on inflectional processing (e.g., Baayen & Moscoso del Prado Martín, 2005; Hare, Ford, & Marslen-Wilson, 1999; Hay, 2001; Sereno & Jongman, 1997; Tabak, Schreuder, & Baayen, 2005; Vitevich & Luce, 1999; Vitevich & Luce, 2005).

In the Words and Rules and declarative/procedural frameworks, such effects have led to the proposal that regular inflections may also be lexically stored and accessed as full forms if they are highly frequent (e.g., Pinker & Ullman, 2002). By contrast, our proposal stresses the automaticity of the segmentation process, which is triggered for any potentially inflected form. In this respect, the account advocated here is more reminiscent of Taft and Forster’s claims for an early, obligatory process of prefix stripping in visual word recognition (Taft & Forster, 1975) – though since the focus of the two accounts is very different, it is hard to compare them directly.

A direct comparison with the Words and Rules and declarative/procedural accounts is also difficult, since their predictions about relative processing times for morphologically simplex and complex words tend to focus on production (see e.g., Pinker and Ullman (2002)). They claim that the retrieval of an irregular form will block the formation of a regularly inflected form in production. If this blocking process transfers to word recognition, it would predict that only real inflected forms undergo decompositional processing. If so, pseudo-inflected forms (and irregulars with the rhyme pattern) should group with monomorphemic forms, but this is not corroborated by our findings.

In terms of connectionist approaches to speech comprehension, the current results have both specific and general implications. First, the results here are inconsistent with the perceptual difficulty account proposed by McClelland, Patterson and associates (e.g., McClelland & Patterson, 2003) to explain apparent selective impairments in processes involving regular inflectional morphology. This account attributes patients’ difficulties with such forms to the additional “phonological complexity and perceptual subtlety” of words ending in coronal obstruents such as [t] and [d]. In the current research we see no evidence for greater processing demands associated with the presence of coronality or obstruency *per se*. Increases in response time and error rate are only seen when these phonemic elements occur in the wider context of the inflectional rhyme pattern, indicating that it is the broader functional context that is relevant here. This broader context seems to have specific morpho-phonological and morpho-syntactic properties, which are both aspects of lexical processing systems that are assigned epiphenomenal status in current connectionist thinking.

These difficulties for the McClelland and Patterson account do not, of course, mean that connectionist accounts cannot be proposed which do incorporate the notion of the IRP, so that the statistical regularities associated with its distribution in English are used to influence network performance. In so far as these regularities have a signalling function that is specifically morphological in nature (as indicated by current results), then such augmented connectionist models would also need some way of capturing these higher-order properties. One way of doing so might be via the morphological sub-regularities that are seen to emerge in some recent models (Davis, van Casteren, & Marslen-Wilson, 2003; Plaut & Gonnerman, 2000; Rueckl & Raveh, 1999). It is less clear how the behaviour of the pseudo-regular pairs could be captured, since semantically they are like the noninflected word pairs, but nonetheless group with the real inflected pairs in the present experiment (though see Plaut and Gonnerman (2000)).

More generally, however, even if a connectionist learning model is implemented that is able to capture the pattern of IRP effects observed here (and in earlier research), this does not mean that we are also obliged to take on board the claims of such a model for the functional architecture of the language processing system. Claims for a single system architecture are not consistent with the extensive evidence that has now accumulated for a more complex and differentiated neuro-biological substrate for human language, especially where morpho-syntactic functions are concerned (for reviews see Marslen-Wilson (2007); Marslen-Wilson and Tyler (2007); Tyler and Marslen-Wilson (2008)). There is indeed room – if not necessity – for statistical learning models as part of an explanatory neuro-cognitive theory, but only if appropriately related to a processing architecture that is neuro-biologically plausible.

The contribution of this paper, in summary, is that it takes our understanding of the language processing system to a different level of specificity, forcing us to be more explicit about the conditions in which we expect certain phonological and morpho-phonological segmentation processes to occur, and how they may impinge on each other. Clearly, a strictly modular interpretation of a dual mechanism account would not have the flexibility to accommodate the multiple interacting factors that play a role during the retrieval of word forms, where some factors would appear to affect all forms regardless of their morphological status, but others uniquely surface when potentially inflected forms are encountered. Rather, as Bybee and McClelland (2005, p399) point out, the processing of these items must use general and specific information simultaneously, without presupposing that they are mutually exclusive. Whatever its underlying computational properties, the processing system will have to be able to handle morpho-phonologically cued information about morphological complexity and more direct mapping between incoming form and lexical representation, while allowing for phonological, semantic and lexical factors to take effect.

Such conflicting pressures on the processing system could perhaps be insightfully considered in an optimality theoretic framework, as suggested by Burzio (2002), but as this study shows, the account will have to allow for reference to the morphological properties of words (e.g., in stratal optimality theory; Bermúdez-Otero, 1999; Kiparsky, 2000). The probabilistic nature of various factors in inflectional processing emphasised by Baayen and Moscoso del Prado Martín (2005), for instance, could also be represented if the conflicting constraints are assumed to be probabilistic in nature (e.g., Boersma and Hayes (2001)). However, regardless of the framework chosen, our study shows that the account will have to be informed by a more systematic examination of the scope of morpho-phonological effects in various inflectional paradigms in typologically different languages.

## Figures and Tables

**Table:** 

Real regular past tense in /t/ and /d/					Nonwords in /t/ and /d/			
	Word 1	Word 2	Allomorph	CV struct	Word 1	Word 2	Ending	CV struct
1	prayed/preyed	pray/prey	d	CCVC	cayed	cay	d	CVC
2	plied	ply	d	CCVC	gared	gare	d	CVC
3	laid	lay	d	CVC	geed	ghee	d	CVC
4	thawed	thaw/thor	d	CVC	miered	mier	d	CVC
5	paid	pay	d	CVC	kied	kie	d	CVC
6	bowed (au)	bow (au)	d	CVC	kushed	kush	t	CVCC
7	wooed	woo	d	CVC	larned	larn	d	CVCC
8	filled	fill	d	CVCC	meast	miece	t	CVCC
9	rubbed	rub	d	CVCC	minned	min	d	CVCC
10	warned	warn/worn	d	CVCC	parft	parf	t	CVCC
11	joined	join	d	CVCC	foped	fope	t	CVCC
12	raised	raise	d	CVCC	dapped	dap	t	CVCC
13	blessed	bless	t	CCVCC	hessed	hess	t	CVCC
14	clapped	clap	t	CCVCC	rawled	rawl	d	CVCC
15	thrashed	thrash	t	CCVCC	soined	soin	d	CVCC
16	wiped	wipe	t	CVCC	wobbed	wob	d	CVCC
17	soaked	soak	t	CVCC	gubbed	gub	d	CVCC
18	seeped	seep	t	CVCC	keered	keer	d	CVCC
19	coped	cope	t	CVCC	boaked	boak	t	CVCC
20	gaped	gape	t	CVCC	bloud	blou	d	CCVC
21	pushed	push	t	CVCC	chayed	chay	d	CCVC
22	washed	wash	t	CVCC	starced	starce	t	CCVCC
23	ceased	cease	t	CVCC	stessed	stess	t	CCVCC
24	pierced	pierce	t	CVCC	jiped	jipe	t	CVCC


**Table:** 

Noncoronal in /p/ and /k/					Nonwords in /p/ and /k/			
	Word 1	Word 2	Ending	CV struct	Word 1	Word 2	Ending	CV struct
1	peak/peek	pea/pee	k	CVC	bjupe	bew	p	CCVC
2	bark	bar/baa	k	CVC	clope	clow	p	CCVC
3	perk	purr	k	CVC	grulp	grull	p	CCVCC
4	mark	mar/ma	k	CVC	qump	qum	p	CCVCC
5	whelk	well	k	CVCC	plamp	plam	p	CCVCC
6	bank	bang	k	CVCC	fupe	foo	p	CVC
7	milk	mill	k	CVCC	rark	rar	k	CVC
8	hulk	hull	k	CVCC	gurk	gur	k	CVC
9	silk	sill	k	CVCC	sark	sar	k	CVC
10	wink	wing	k	CVCC	durk	durr	k	CVC
11	rink	ring/wring	k	CVCC	pelk	pell	k	CVCC
12	kink	king	k	CVCC	tulk	tull (hull)	k	CVCC
13	troop/troupe	true	p	CCVC	fink	fing	k	CVCC
14	slope	slow/sloe	p	CCVC	mank	mang	k	CVCC
15	clamp	clam	p	CCVCC	bink	bing	k	CVCC
16	plump	plum/plumb	p	CCVCC	lilk	lill	k	CVCC
17	cramp	cram	p	CCVCC	hink	hing	k	CVCC
18	soap	sew/so	p	CVC	shulk	shull	k	CVCC
19	hemp	hem	p	CVCC	famp	fam	p	CVCC
20	lamp	lamb	p	CVCC	wump	wum	p	CVCC
21	damp	dam/damn	p	CVCC	lemp	lem	p	CVCC
22	hump	hum	p	CVCC	simp	sim	p	CVCC
23	rump	rum	p	CVCC	mamp	mam	p	CVCC
24	gulp	gull	p	CVCC	bamp	bam	p	CVCC

**Table:** 

Nonpseudo-regulars in /t/					Nonwords in /t/			
	Word 1	Word 2	Ending	CV struct	Word 1	Word 2	Ending	CV struct
1	saint	sane	t	CVCC	twight	twy	t	CCVC
2	cult	cull	t	CVCC	steet	stee	t	CCVC
3	tint	tin	t	CVCC	prunt	prun	t	CCVCC
4	dent	den	t	CVCC	prilt	prill	t	CCVCC
5	bolt	bowl	t	CVCC	crilt	crill	t	CCVCC
6	punt	pun	t	CVCC	nart	nar	t	CVC
7	rent	wren	t	CVCC	lart	lar	t	CVC
8	hilt	hill	t	CVCC	shaint	shane	t	CVC
9	guilt	gill	t	CVCC	shayt	shay	t	CVC
10	dint	din	t	CVCC	deet	dee	t	CVC
11	tilt	till	t	CVCC	haint	hain	t	CVCC
12	pint	pine	t	CVCC	sult	sull	t	CVCC
13	belt	bell	t	CVCC	rint	rin	t	CVCC
14	tent	ten	t	CVCC	nent	nen	t	CVCC
15	light	lie	t	CVC	ghent	ghen	t	CVCC
16	cart	car	t	CVC	nint	nin	t	CVCC
17	neat	knee	t	CVC	sant	san	t	CVCC
18	port	pour/paw/poor	t	CVC	kint	kine	t	CVCC
19	bait/bate	bay	t	CVC	rult	rull	t	CVCC
20	stilt	still	t	CCVCC	yilt	yill	t	CVCC
21	quilt	quill	t	CCVCC	ghelt	ghell	t	CVCC
22	stunt	stun	t	CCVCC	shent	shen	t	CVCC
23	slight	sly	t	CCVC	shilt	shill	t	CVCC
24	start	star	t	CCVC	lunt	lun	t	CVCC

**Table:** 

Pseudo-regulars in /t/ and /d/				
	Word 1	Word 2	Ending	CV struct
1	ford	for	d	CVC
2	wand	wan	d	CVCC
3	wide	why	d	CVC
4	field	feel	d	CVCC
5	tweed	twee	d	CCVC
6	cord	core	d	CVC
7	beard	beer	d	CVC
8	weird	weir	d	CVC
9	gold	goal	d	CVCC
10	mild	mile	d	CVCC
11	proud	prow	d	CCVC
12	wind	win	d	CVCC
13	cold	coal	d	CVCC
14	bald	ball/bawl	d	CVCC
15	fund	fun	d	CVCC
16	mould	mole	d	CVCC
17	rift	riff	t	CVCC
18	graft	graph	t	CCVCC
19	chest	chess	t	CVCC
20	bust	bus	t	CVCC
21	fast	farce	t	CVCC
22	crest	cress	t	CCVCC
23	deft	deaf	t	CVCC
24	tuft	tough	t	CVCC

**Table:** 

Present in /s/ and /z/					Nonwords in /s/ and /z/			
	Word 1	Word 2	Allomorph	CV struct	Word 1	Word 2	Ending	CV struct
1	gloats	gloat	s	CCVCC	groys	groy	z	CCVC
2	sniffs	sniff	s	CCVCC	graws	graw	z	CCVC
3	flips	flip	s	CCVCC	plocks	plock	s	CCVCC
4	licks	lick	s	CVCC	throcks	throck	s	CCVCC
5	mocks	mock	s	CVCC	glips	glip	s	CCVCC
6	tucks	tuck	s	CVCC	fies	fie	z	CVC
7	picks	pick	s	CVCC	koes	ko	z	CVC
8	bakes	bake	s	CVCC	sares	sare	z	CVC
9	pokes	poke	s	CVCC	tays	tay	z	CVC
10	wraps/raps	wrap/rap	s	CVCC	tause	tau	z	CVC
11	reaps	reap	s	CVCC	pives	pive	z	CVCC
12	rips	rip	s	CVCC	baves	bave	z	CVCC
13	stirs	stir	z	CCVC	cuvs	cuv	z	CVCC
14	stows	stow	z	CCVC	dags	dag	z	CVCC
15	dies/dyes	die/dye	z	CVC	dapes	dape	z	CVCC
16	mows	mow	z	CVC	bips	bip	s	CVCC
17	gnaws	gnaw/nor	z	CVC	pakes	pake	s	CVCC
18	shows	show	z	CVC	soats	soat	s	CVCC
19	coos/coups	coo	z	CVC	bots	bot	s	CVCC
20	lives	live	z	CVCC	gucks	guck	s	CVCC
21	fails	fail	z	CVCC	bicks	bick	s	CVCC
22	begs	beg	z	CVCC	dakes	dake	s	CVCC
23	saves	save	z	CVCC	hicks	hick	s	CVCC
24	nags	nag	z	CVCC	lats	lat	s	CVCC

**Table:** 

Plural in /s/ and /z/				
	Word 1	Word 2	Ending	CV struct
1	threats	threat	s	CCVCC
2	grapes	grape	s	CCVCC
3	snouts	snout	s	CCVCC
4	lips	lip	s	CVCC
5	debts	debt	s	CVCC
6	cats	cat	s	CVCC
7	shirts	shirt	s	CVCC
8	lakes	lake	s	CVCC
9	goats	goat	s	CVCC
10	hips	hip	s	CVCC
11	shots	shot	s	CVCC
12	paths	path	s	CVCC
13	dunes	dune	z	CCVC
14	globes	globe	z	CCVC
15	cows	cow	z	CVC
16	psalms	psalm	z	CVC
17	doors	door	z	CVC
18	thorns	thorn	z	CVC
19	whims	whim	z	CVC
20	birds	bird	z	CVCC
21	cones	cone	z	CVCC
22	barns	barn	z	CVCC
23	meals	meal	z	CVCC
24	yards	yard	z	CVCC

**Table:** 

Real regular past in /Id/					Nonword /Id/			
	Word 1	Word 2	Allomorph	CV struct	Word 1	Word 2	Ending	CV struct
1	greeted	greet	Id	CCVC(VC)	scaded	scade	Id	CCVC
2	fretted	fret	Id	CCVC(VC)	snaiting	snait	Id	CCVC
3	flaunted	flaunt	Id	CCVCC(VC)	clended	clend	Id	CCVCC
4	jilted	jilt	Id	CCVCC(VC)	flanted	flant	Id	CCVCC
5	scolded	scold	Id	CCVCC(VC)	groasted	groast	Id	CCVCC
6	knitted	knit/nit	Id	CVC(VC)	ketted	ket	Id	CVC
7	waded	wade/weighed	Id	CVC(VC)	reeted	reet	Id	CVC
8	waited/weighted	wait/weight	Id	CVC(VC)	yitted	yit	Id	CVC
9	faded	fade	Id	CVC(VC)	bodded	bod	Id	CVC
10	nodded	nod	Id	CVC(VC)	saded	sade	Id	CVC
11	lasted	last	Id	CVCC(VC)	maisted	maist	Id	CVCC
12	melted	melt	Id	CVCC(VC)	woasted	woast	Id	CVCC
13	welded	weld	Id	CVCC(VC)	waunted	waunt	Id	CVCC
14	wanted	want/wont	Id	CVCC(VC)	pilded	pild	Id	CVCC
15	basted	baste/based	Id	CVCC(VC)	bulted	bult	Id	CVCC
16	mended	mend	Id	CVCC(VC)	relted	relt	Id	CVCC
17	wilted	wilt	Id	CVCC(VC)	wended	wend	Id	CVCC
18	wielded	wield	Id	CVCC(VC)	sested	sest	Id	CVCC
19	gilded	gild/guild	Id	CVCC(VC)	walded	wald	Id	CVCC
20	wafted	waft	Id	CVCC(VC)	munted	munt	Id	CVCC
21	pelted	pelt	Id	CVCC(VC)	banted	bant	Id	CVCC
22	ranted	rant	Id	CVCC(VC)	bafted	baft	Id	CVCC
23	boasted	boast	Id	CVCC(VC)	gielded	gield	Id	CVCC
24	shunted	shunt	Id	CVCC(VC)	milted	milt	Id	CVCC

**Table:** 

Progressive aspect					Nonword			
	Word 1	Word 2	Allomorph	CV struct	Word 1	Word 2	Ending	CV struct
1	drowning	drown		CCVC(VC)	cleading	clead	ing	CCVC
2	pleading	plead		CCVC(VC)	cleeming	cleem	ing	CCVC
3	cleansing	cleanse		CCVCC(VC)	ploaxing	ploax	ing	CCVCC
4	clanking	clank		CCVCC(VC)	slending	slend	ing	CCVCC
5	slinking	slink		CCVCC(VC)	frinking	frink	ing	CCVCC
6	hurting	hurt		CVC(VC)	bowning	bown	ing	CVC
7	seeming	seem/seam		CVC(VC)	dading	dade	ing	CVC
8	lurking	lurk		CVC(VC)	surking	surk	ing	CVC
9	teasing	tease		CVC(VC)	measing	mease	ing	CVC
10	leaning	lean		CVC(VC)	werting	wert	ing	CVC
11	yanking	yank		CVCC(VC)	fanking	fank	ing	CVCC
12	solving	solve		CVCC(VC)	folving	folve	ing	CVCC
13	thanking	thank		CVCC(VC)	panking	pank	ing	CVCC
14	founding	found		CVCC(VC)	lounding	lound	ing	CVCC
15	resting/wresting	rest/wrest		CVCC(VC)	tarsting	tarst	ing	CVCC
16	tending	tend		CVCC(VC)	drending	drend	ing	CVCC
17	bending	bend		CVCC(VC)	sunching	sunch	ing	CVCC
18	coaxing	coax/cokes		CVCC(VC)	rouncing	rounce	ing	CVCC
19	sounding	sound		CVCC(VC)	fasking	fask	ing	CVCC
20	yielding	yield		CVCC(VC)	fifting	fift	ing	CVCC
21	sifting	sift		CVCC(VC)	selving	selve	ing	CVCC
22	fending	fend		CVCC(VC)	loasting	loast	ing	CVCC
23	bouncing	bounce		CVCC(VC)	touncing	tounce	ing	CVCC
24	folding	fold		CVCC(VC)	yixing	yix	ing	CVCC

**Table:** 

Condition		Mean (ms) *different* items^a^	Mean (ms) *same* items^b^
Regular past /t/ /d/	*filled–fill*	967	926
Pseudo past /t/ /d/	*mild–mile*	977	907
[-Cor, −VoiAgree] /k, p/	*lamp–lamb*	827	869
[+Cor, −VoiAgree] /t/	*belt–bell*	823	856
Present /s/ /z/	*fails–fail*	935	889
Plural /s/ /z/	*meals–meal*	935	884
Regular past /Id/	*folded–fold*	809	805
Progressive	*fending–fend*	835	837
Nonword /d/ /t/	*gubbed–gub*	922	899
Nonword /t/ (not past)	*steet–stee*	858	896
Nonword /p/ /k/	*wump–wum*	838	872
Nonword /s/ /z/	*pakes–pake*	956	879
Nonword /Id/	*milted–milt*	832	836
Nonword	*sunching–sunch*	829	815

a Condition: *F*(13, 313) = 12.66, *p* < .001; word 2 duration: *F*(1, 313) = 109.53, *p* < .001. Covariates appearing in the model are evaluated at the following values: duration word 2 = 696.14.

b Condition: *F*(13, 319) = 5.25, *p* < .001; word 2 duration: *F*(1, 319) = 239.66, *p* < 001. Covariates appearing in the model are evaluated at the following values: duration word 2 = 772.03.

**Table 1:** Overview of experimental conditions

		**Real word conditions**	**Nonword conditions**
Regular past tense baseline	1	Regular past in /t/& /d/ [+Coronal, +Voice Agreement] *filled – fill*	in /t/ and /d/ *gubbed - gub*

Coronality and voicing	2	Pseudo past in /t/ & /d/ [+Coronal, +Voice Agreement] *mild – mile*	also *gubbed - gub*
	3	[+Coronal, -VoiceAgreement] in /t/ *belt - bell*	in /t/ *steet - stee*
	4	[-Coronal, -VoiceAgreement] in /p/ & /k/ *lamp - lamb*	in /p/ and /k/ *wump - wum*

Inflectional paradigm (Manner of articulation)	5	Present tense in /s/ & /z/ [+Coronal, +Voice Agreement] *fails - fail*	in /s/ and /z/ *pakes - pake*
	6	Plural in /s/ & /z/ [+Coronal, +Voice Agreement] *meals - meal*	also *pakes - pake*

Syllabicity	7	Regular past in /id/ *folded - fold*	in **/**id**/***milted – milt*
	8	Progressive aspect *fending – fend*	in *sunching - sunch*

**Table 2:** Mean duration of the second word for each condition

Condition		Duration word 2 in ms					
		Real words			Nonwords		
		Overall	Same	Diff.	Overall	Same	Diff.
Real regular past	*filled–fill*	741	765	717	733	752	713
Pseudo past in /t/ /d/	*mild–mile*	721	753	689			
[−Cor, −Voice] in /p/ /k/	*lamp–lamb*	683	707	658	661	680	641
[+Cor, −Voice] in /t/	*belt–bell*	665	697	632	693	713	672
Present tense in /s/ /z/	*fails–fail*	725	796	654	703	761	645
Plural in /s/ and /z/	*meals–meal*	761	813	709			
Regular past in /Id/	*folded–fold*	754	819	689	784	822	745
Progressive aspect	*fending–fend*	810	851	768	846	879	812

**Table 3:** Descriptive lexical statistics by condition: median lemma frequencies per million, summed word form frequencies per million, and familiarity and imageability ratings (word 2 medians reported for the *different* pairs)

Condition		Word 1					Word 2				
		Lemma		Wf sum	Fam	Imag	Lemma		Wf sum	Fam	Imag
		noun	verb				noun	verb			
Real regular past	*filled–fill*	0	37	14	500	415	0	37	13	504	441
Pseudo past in /t/ /d/	*mild–mile*	5	0	14	539	504	5	0	14	506	505
[−Cor, −Voice] in /p/ /k/	*lamp–lamb*	3	3	3	435	463	5	3	10	469	467
[+Cor, −Voice] in /t/	*belt–bell*	10	1	9	481	449	9	1	10	517	447
Present tense in /s/ /z/	*fails–fail*	0	14	1	469	400	0	14	1	484	400
Plural in /s/ and /z/	*meals–meal*	37	0	13	510	549	37	0	10	510	569
Regular past in /Id/	*folded–fold*	0	7	2	491	398	0	7	5	491	408
Progressive aspect	*fending–fend*	0	22	6	429	384	0	22	7	468	398

**Table 4:** Harmonic mean reaction times and error rates for *same* and *different* judgments: (1) grand mean, (2) *same* items, and (3) *different* items, by condition

Condition		Harmonic mean RT (ms)			Error proportion (%)		
		Overall	Same	Diff.	Overall	Same	Diff.
Regular past /t/ /d/	*filled–fill*	949	922	979	4.0	4.1	3.9
Pseudo past /t/ /d/	*mild–mile*	932	895	973	5.5	4.2	6.9
[−Cor, −VoiAgree] /k/ /p/	*lamp–lamb*	821	831	812	1.6	2.3	0.9
[+Cor, −VoiAgree] /t/	*belt–bell*	806	813	799	2.5	1.9	3.1
Present /s/ /z/	*fails–fail*	909	905	914	2.9	4.0	1.7
Plural /s/ /z/	*meals–meal*	927	912	942	3.8	3.5	4.0
Regular past /Id/	*folded–fold*	819	832	806	2.0	2.5	1.5
Progressive	*fending–fend*	876	887	866	3.1	2.5	3.8
Nonword /d/ /t/	*gubbed–gub*	908	886	932	3.1	1.5	4.8
Nonword /t/ (not past)	*steet–stee*	853	858	848	3.2	4.6	1.9
Nonword /p/ /k/	*wump–wum*	816	817	815	3.2	4.2	2.2
Nonword /s/ /z/	*pakes–pake*	900	872	929	3.6	3.1	4.2
Nonword /Id/	*milted–milt*	860	866	853	3.8	5.0	2.6
Nonword	*sunching–sunch*	879	879	879	2.2	3.3	1.0

**Table 5:** Regression analysis of reaction time including all interactions with judgment type: standardized coefficients with *t* values and significance levels

	St. Coeff. Beta	*t*	Sig.
*Level 1: regressors*			
(Constant)		58.89	*p* < .001
Duration word 2	−0.63	−18.78	*p* < .001
Morphological structure	0.00	−0.09	*p* = .92
Word type	0.02	0.54	*p* = .59
Rhyme pattern	−0.35	−6.79	*p* < .001
Place (coronal or not)	0.03	0.71	*p* = .48
Voice	0.07	1.53	*p* = .13
Syllabicity	0.41	9.64	*p* < .001
Manner (stop or not)	−0.05	−1.21	*p* = .23
Judgment type	−0.71	−3.36	*p* < .001

*Level 2: Interactions with judgment type*			
Duration word 2 × judgment type	0.06	1.23	*p* = .22
Morphological structure × judgment type	−0.03	−0.75	*p* = .45
Word type × judgment type	−0.24	−4.33	*p* < .001
Rhyme pattern × judgment type	−0.02	−0.43	*p* = .67
Place (coronal or not) × judgment type	0.10	2.23	*p* < .05
Voice × judgment type	0.04	0.92	*p* = .36
Syllabicity × judgment type	0.04	1.04	*p* = .30
Manner (stop or not) × judgment type	0.61	2.98	*p* < .001

## Footnotes

1 Word 1: lemma frequency noun *F*(7, 371) = 6.83, *p* < .001, lemma frequency verb *F*(7, 371) = 4.01, *p* < .001, word form frequency *F*(7, 371) = 2.74, *p* < .01, marginal for familiarity *F*(7, 371) = 2.00, *p* = .054, imageability *F*(7, 371) = 6.70, *p* < .001; word 2: lemma frequency noun *F*(7, 371) = 6.84, *p* < .001], lemma frequency verb *F*(7, 371) = 3.20, *p* < .01, word form frequency *F*(7, 371) = .92, *p* = .49, imageability *F*(7, 371) = 6.54, *p* < .001, familiarity *F*(7, 371) = .59, *p* = .77.

2 The item with the 85% error score was an outlier: only 5 other items had error rates above 25% (1 at 35% and 4 at 25%). The missing items were two regular past *different* items, one regular past *same* item, one present tense *different* item, one noncoronal /k/ /p/ *different* item, two *different* items from the nonwords in /t/ and /d/, one *different* item from the nonwords in /k/ and /p/, and one *different* item from the nonwords in /Id/.

3 The same pattern of results obtained when we included word 2 duration as a covariate in an analysis of variance with between-subject factors condition (14 levels) and judgment type (2 levels) [condition: *F*(13, 633) = 15.81, *p* < .001, judgment type: *F*(1, 633) = 60.05, *p* < .001, condition × judgment type: *F*(13, 633) = 2.51, *p* < .01, word 2 duration: *F*(1, 633) = 324.07, *p* < .001].

4 For word 1: lemma frequency noun *r*(379) = −.04, *p* = .47, lemma frequency verb *r*(379) = −.07, *p* = .15, word form frequency *r*(379) = −.06, *p* = .21, familiarity *r*(379) = −.01, *p* = .43, imageability *r*(379) = −.08, *p* = .13; for word 2: lemma frequency noun *r*(379) = −.01, *p* = .93, lemma frequency verb *r*(379) = −.08, *p* = .13, word form frequency *r*(379) = −.01, *p* = .87, familiarity *r*(379) = −.03, *p* = .62, imageability *r*(379) = −.08, *p* = .10. The correlation is negative for inverse transformed reaction times.

5 The significant interaction between judgment type and word type can be attributed to smaller *differences* between *same* and *different* items for nonwords than for real words; the interaction with place of articulation reflects equally fast responses to *same* and *different* items for the /k/ and /p/ conditions compared to differences between the judgement types in the other conditions, and similarly, the interaction with manner reflects a fairly consistent difference between *same* and *different* responses for all items in /s/ and /z/, but varied differences between judgment types for the other conditions.

6 Since the values were estimated in a different analysis, they are different from those in Fig. 1 for all conditions, even when no items were removed from the condition (e.g., [+Cor, −VoiceAgreement] /t/). Note that there are unequal numbers of items in the conditions in Fig. 2.

7 Word 2 duration *p* = .24; for word 1: lemma frequency noun *p* = .59, lemma frequency verb *p* = .69, word form frequency *p* = .18, familiarity *p* = .67, imageability *p* = .19; for word 2: lemma frequency noun *p* = .97, lemma frequency verb *p* = .54, word form frequency *p* = .36, familiarity *p* = .85, imageability *p* = .56.

8 There is ample evidence that inflectional morphemes are not only extracted in the same–different task used here, but in many other processing conditions which do not encourage participants to focus on the endings of the items (e.g., Lehtonen, Vorobyev, Hugdahl, Tuokkola, & Laine 2006; Tyler et al. 2002a; Wurm, 1997).
